# Genetic variants in the Hippo pathway predict biochemical recurrence after radical prostatectomy for localized prostate cancer

**DOI:** 10.1038/srep08556

**Published:** 2015-02-24

**Authors:** Chao-Yuan Huang, Shu-Pin Huang, Victor C. Lin, Chia-Cheng Yu, Ta-Yuan Chang, Shin-Hun Juang, Bo-Ying Bao

**Affiliations:** 1 Department of Urology, National Taiwan University Hospital, College of Medicine, National Taiwan University, Taipei, Taiwan; 2 Department of Urology, Kaohsiung Medical University Hospital, Kaohsiung, Taiwan; 3 Department of Urology, Faculty of Medicine, College of Medicine, Kaohsiung Medical University, Kaohsiung, Taiwan; 4 Department of Urology, E-Da Hospital, Kaohsiung, Taiwan; 5 School of Medicine for International Students, I-Shou University, Kaohsiung, Taiwan; 6 Division of Urology, Department of Surgery, Kaohsiung Veterans General Hospital, Kaohsiung, Taiwan; 7 Department of Urology, School of Medicine, National Yang-Ming University, Taipei, Taiwan; 8 Department of Pharmacy, Tajen University, Pingtung, Taiwan; 9 Department of Occupational Safety and Health, China Medical University, Taichung, Taiwan; 10 Department of Pharmacy, China Medical University, Taichung, Taiwan; 11 Sex Hormone Research Center, China Medical University Hospital, Taichung, Taiwan; 12 Department of Nursing, Asia University, Taichung, Taiwan

## Abstract

While localized prostate cancer is potentially curative, many patients still show biochemical recurrence (BCR) after curative treatments such as radical prostatectomy (RP). The Hippo pathway has recently been shown to be an evolutionarily conserved regulator of tissue growth, and its perturbation can trigger tumorigenesis. We hypothesize that genetic variants of the Hippo pathway may influence clinical outcomes in localized prostate cancer patients. We genotyped 53 tagging single-nucleotide polymorphisms (SNPs) from seven core Hippo pathway genes in 246 localized prostate cancer patients treated with RP. Kaplan-Meier analysis and Cox proportional hazard models were utilized to identify significant SNPs that correlated with BCR. For replication, five associated SNPs were genotyped in an independent cohort of 212 patients. After adjusting for known clinicopathologic factors, the association between *STK3* rs7827435 and BCR (*P* = 0.018) was replicated in the second stage (*P* = 0.026; *P*_combined_ = 0.001). Additional integrated *in silico* analysis provided evidence that rs7827435 affects *STK3* expression, which in turn is significantly correlated with tumor aggressiveness and patient prognosis. In conclusion, genetic variants of the Hippo pathway contribute to the variable outcomes of prostate cancer, and the discovery of these biomarkers provides a molecular approach for prognostic risk assessment.

Prostate cancer continues to be one of the most frequently diagnosed malignancies in men. A large number of the new cases are being detected in younger patients, often presenting in a clinically localized stage^1^. Radical prostatectomy (RP) is widely performed as the definitive treatment for such patients. Although several risk stratification systems are currently in use for the management of prostate cancer, including those based on prostate-specific antigen (PSA), Gleason score, or the tumor stage, up to 40% of the patients undergoing RP eventually experience biochemical recurrence (BCR) and need additional treatment^2^,^3^. Therefore, new genres of biomarkers are needed to facilitate clinical decisions in the management of prostate cancer.

Recent studies have established that the genes of the Hippo signaling pathway play a pivotal role in controlling organ size through regulation of cell proliferation and apoptosis^4^. The core of the Hippo pathway comprises a kinase cassette that consists of several serine/threonine kinases, mammalian STE20-like protein kinase 1 and 2 (MST1 and MST2, also known as STK3), and large tumor suppressors 1 and 2 (LATS1/2), together with the adaptor proteins Salvador homologue 1 (SAV1) and MOB kinase activator 1A and 1B (MOB1A/1B). The MST1/2 and SAV1 complex can phosphorylate and activate the LATS1/2 and MOB1A/1B complex^5^. In turn, activated LATS1/2 can directly interact with and phosphorylate the Yes-associated protein (YAP1) and the WW domain containing transcription regulator 1 (WWTR1)^6^. Phosphorylation of the YAP1/WWTR1 proteins represses their activity by creating 14-3-3 binding sites, causing cytoplasmic accumulation and subsequent ubiquitin-mediated proteolysis^7^. The YAP1/WWTR1 proteins promote tissue growth by binding transcription factors from the TEA domain- and SMAD families, in order to regulate genes involved in proliferation, differentiation, and development^8^. The dysregulation of the Hippo pathway has been reported in a broad range of human cancers, including prostate^9^, and it often correlates with poor patient prognosis^10^.

In this study, we evaluated whether genetic variations within the Hippo pathway correlate with the BCR-free survival in two independent cohorts of localized prostate cancer patients treated with RP.

## Results

### Patient characteristics and treatment outcome

Patient characteristics of the discovery and replication cohorts are described in Table 1. The median follow-up times were 50 and 60 months; 75 (30.5%) and 109 (51.4%) patients experienced BCR in the discovery and replication cohorts, respectively. PSA at diagnosis, pathologic Gleason score, and stage, were all significantly associated with BCR (*P* < 0.001).

### Association between the Hippo pathway single-nucleotide polymorphisms (SNPs) and BCR

**Figure 1:**
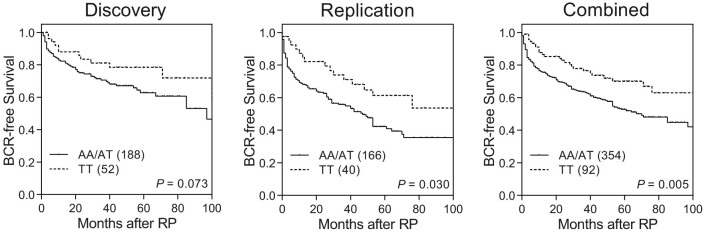
Kaplan-Meier survival curves of BCR-free survival by *STK3* rs7827435 genotypes for localized prostate cancer patients receiving RP in the discovery cohort (left), replication cohort (middle), and combined analysis (right). Numbers in parentheses indicate the number of patients.

We used a two-step approach to analyze 53 SNPs in a discovery phase and validate the associations (five SNPs showed association with BCR, *P* < 0.05, in the discovery phase) in another independent cohort, for a total of 458 men with organ-confined prostate cancer. Results of the discovery phase are reported in Supplementary Table 1, while results of the combined analysis of the discovery and replication phases are reported in Table 2. Only *STK3* rs7827435 showed significant correlation with a decreased risk of recurrence in both the discovery and replication cohorts (*P* ≤ 0.048), and upon combined analysis [hazard ratio (HR) 0.56, 95% confidence interval (CI) 0.38–0.84, *P* = 0.005; Table 2 and Figure 1]. To assess the predictive effects of *STK3* rs7827435 beyond the currently used clinical variables, we performed a multivariate analysis, adjusting for age, PSA at diagnosis, pathologic Gleason score, and stage. After adjusting for these predictors, the association remained significant in the discovery cohort (HR 0.45, 95% CI 0.23–0.87, *P* = 0.018; Table 2), the replication cohort (HR 0.53, 95% CI 0.30–0.93, *P* = 0.026), and upon combined analysis (HR 0.49, 95% CI 0.32–0.76, *P* = 0.001).

### Functional analyses of the *STK3* rs7827435 locus

**Figure 2:**
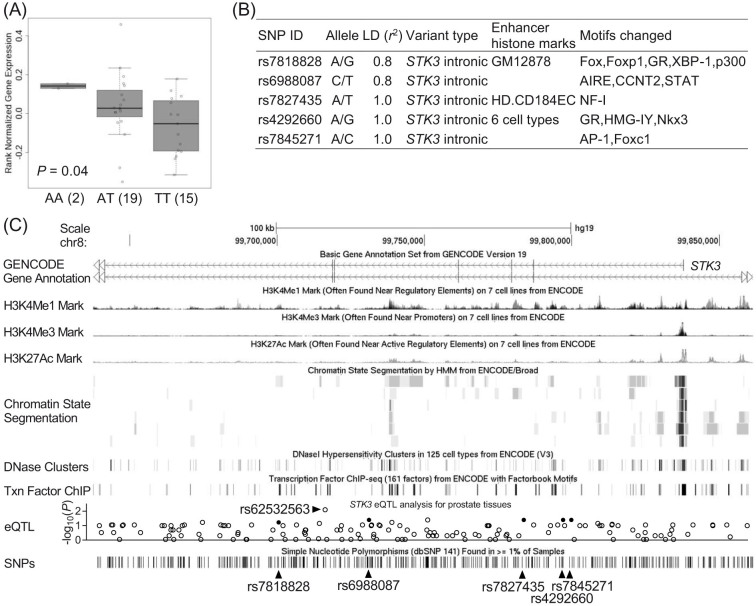
Summary of the functional analyses for the *STK3* rs7827435 locus. (A) *STK3* shows significant eQTL association with rs7827435 genotype in prostate tissues (GTEx data set). Numbers in parentheses indicate the number of cases. (B) Regulatory annotation of variants within the LD block containing *STK3* rs7827435. In the LD block with the lead SNP rs7827435, ENCODE data showed evidence of enhancer elements coinciding with rs7827435 and linked variants in GM12878 B-lymphoblastoid cells, HD.CD184EC hESC-derived CD184+ endoderm cultured cells, and several additional cell types. In addition, multiple regulatory motifs are predicted to be affected. (C) Expanded view of the ENCODE and GTEx data for the LD block containing the *STK3* rs7827435. The H3K4Me1, H3K4Me3, and H3K27Ac tracks show the genome-wide levels of enrichment of the mono-methylation of lysine 4, tri-methylation of lysine 4, and acetylation of lysine 27 of the H3 histone protein, as determined by the ChIP-seq assays. These levels are thought to be associated with enhancer and promoter regions. Chromatin State Segmentation track displays chromatin state segmentations by integrating ChIP-seq data using a Hidden Markov Model for GM12878 B-lymphoblastoid cells, K562 leukemia cells, HepG2 hepatocellular carcinoma cells, HMEC normal mammary epithelial cells, NHEK epidermal keratinocytes, and NHLF normal lung fibroblast cells. The chromatin state regions predicted for promoters and enhancers are highlighted. DNase clusters track shows DNase hypersensitivity areas. Tnx Factor track shows regions of transcription factor binding of DNA, as assayed by ChIP-seq experiments. The results of *STK3* gene-centric eQTL analysis for prostate tissues using GTEx data are visualized as a regional plot. Closed circles highlight where rs7827435 and its linked SNPs are placed.

We investigated whether rs7827435 was associated with differential expression of the *STK3*, as a preliminary assessment of the putative functional role of the SNP. The Genotype-Tissue Expression (GTEx) database showed a significant trend for decreased *STK3* mRNA expression in prostate tissues of rs7827435 variant allele (T) carriers (*P* = 0.04, Figure 2A). Functional annotations from the Encyclopedia of DNA Elements (ENCODE) and Roadmap Epigenomics data are shown for all correlated variants within the linkage disequilibrium (LD) block (*r*^2^ > 0.8) of rs7827435 in Figures 2B and 2C. The rs7827435 SNP and two additional rs7827435-linked SNPs, rs7818828 and rs4292660, are situated at a locus with histone modification patterns that characterize enhancers in several cell types. In addition, multiple regulatory motifs were predicted to be altered by these SNPs (Figure 2B). Together, rs7827435 A to T substitution might affect transcription factor binding, decrease *STK3* mRNA expression, and reduce the risk of prostate cancer recurrence.

### Correlation of *STK3* expression with prostate cancer progression

**Figure 3:**
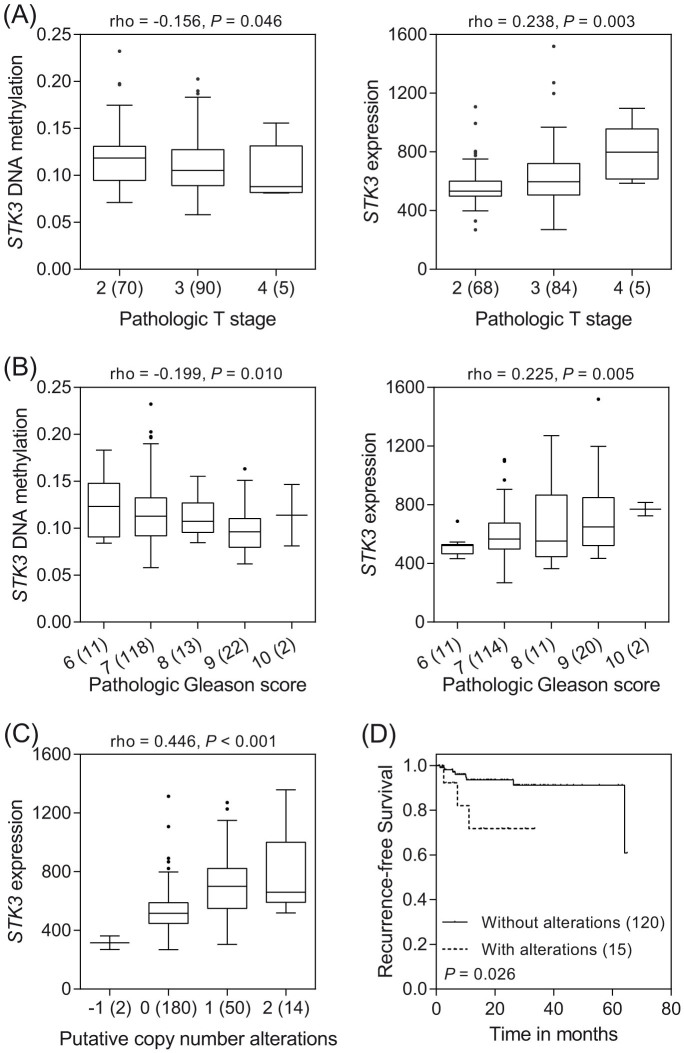
Correlation of *STK3* mRNA expression with prostate cancer progression. The associations between *STK3* expression and prostate cancer aggressiveness were analyzed using TCGA data. More advanced prostate cancers, high pathologic stage (A) and pathologic Gleason score (B), display significantly lower *STK3* DNA methylation and higher *STK3* mRNA expression. Numbers in parentheses indicate the number of patients. rho: Spearman's rank correlation coefficient. P: *P*-value. (C) *STK3* shows higher levels of gene expression in tumors with increased DNA copy number at 8q22. −1: hemizygous deletion; 0: diploid; 1: gain; 2: amplification. (D) Kaplan-Meier curves of recurrence-free survival according to the alterations in *STK3*. Patients were dichotomized with or without *STK3* amplification and mRNA upregulation.

To further confirm the influence of *STK3* on prostate cancer progression, we performed a comprehensive *in silico* evaluation of *STK3* gene expression, DNA methylation, and gene copy number using publicly available The Cancer Genome Atlas (TCGA) datasets. Both *STK3* DNA hypomethylation and mRNA upregulation were significantly correlated with higher pathologic stages and Gleason score tumors (*P* ≤ 0.046, Figure 3A and 3B). Furthermore, gene copy number was also found to be correlated with mRNA expression for *STK3* (*P* < 0.001, Figure 3C). The follow-up of this cohort established that gene amplification and mRNA upregulation of *STK3* were strongly associated with a higher risk of prostate cancer recurrence (*P* = 0.026, Figure 3D).

## Discussion

In this hypothesis-driven association study of seven core Hippo pathway genes, *STK3* was significantly associated with BCR in both the discovery and replication cohorts. We present additional evidence for a role of *STK3* in prostate cancer, as elevated *STK3* gene expression was associated with more aggressive cancers and poorer clinical outcomes. These results support the hypothesis of a link between the genetic variants of the Hippo pathway and prostate cancer progression.

The strongest signal, tagged by rs7827435, is located in a ~ 100-kb LD block covering the 5' region of the *STK3*. According to *STK3* gene-centric expression quantitative trait locus (eQTL) data from GTEx for prostate tissues, the best *STK3* eQTL SNP in this region was rs62532563 (*P* = 0.008; Figure 2C), but it was not genotyped in HapMap and this study. The second best eQTL SNPs for *STK3* were rs7827435 and its linked variants. rs62532563, rs7827435, and rs7827435-linked SNPs are in the same haplotype block, suggesting that the variants in this genomic location might regulate *STK3* expression and lead to the observed phenotype. Functional annotations from the ENCODE and Roadmap Epigenomics data indicate that rs7827435 and two rs7827435-linked SNPs, rs7818828 and rs4292660 (*r*^2^ = 0.8 and 1.0 with rs7827435, respectively), coincide with regions of open chromatin, which probably correspond to the enhancers of *STK3* (Figure 2B and 2C). In addition, rs7827435 is predicted to influence a nuclear factor I (NF-I) binding site, where the minor T allele weakens the predicted affinity relative to the major A allele. eQTL analysis also found that the rs7827435 T allele was associated with reduced *STK3* expression (Figure 2A). Low levels of *STK3* were detected in less aggressive prostate tumors using TCGA dataset (Figure 3A and 3B). Consistent with our data, patients with an rs7827435 TT genotype (low expression group) had a reduced recurrence risk compared with carriers of the major A allele (high expression group; Figure 1 and 3D).

*STK3* is located within chromosome 8q22.2, which shows high relevance with chromosomal aberrations in several types of cancer, including prostate, breast, bladder, and oral squamous cell carcinoma^11^,^12^,^13^,^14^. The gain of 8q is the most common chromosomal alteration detected in castration-resistant and metastatic prostate carcinomas with almost 90% of the advanced tumors^15^,^16^. These data were consistent with our observation that rs7827435 A allele was associated with increased *STK3* expression, which in turn is correlated with poor patient prognosis. However, *STK3* has been best characterized as a tumor suppressor^17^, and its positive role in cancer progression has seldom been reported. In the canonical Hippo pathway, STK3 forms complexes with SAV1, MOBs, and LATSs, resulting in the phosphorylation of LATSs and subsequent transcription of proapoptotic genes^18^. Intriguingly, recent studies have demonstrated that *STK3* is required for optimal cell proliferation in response to mitogen stimulation^19^. Conversely, *STK3* is also required for maximal anticancer drug-induced apoptotic cell death in the same cell types. Other MST family members have been implicated in prostate cancer, including MST1 and MST4, while MST1 levels were found to decline with disease progression^20^, MST4 expression levels were positively correlated with the tumorigenicity of prostate cancer^21^. Therefore, MSTs, including STK3, exert a paradoxical and complicated effect during cancer development, and further investigations are warranted to uncover the mechanistic basis of the effect of the Hippo pathway genes on prostate cancer.

We noted that many Hippo pathway SNPs (one in *MST1*, three in *STK3*, two in *LATS2*, one in *MOB1B*, and six in *WWTR1*; total 13 out of 75) genotyped in our prostate cancer patient cohort were not in Hardy-Weinberg equilibrium (HWE). There are several possible explanations behind this deviation. First, it has been suggested that the deviation from HWE in patient group only might provide support for an association between studied locus and disease^22^. However, we did not take for granted that every deviated SNP in cases indicates a probable genetic association. Second, considering a > 0.99 agreement on genotyping calls across all SNPs assayed in 10 blind duplicate samples, we do not expect a mistyping in our study to cause the deviation. Finally, there might have been a selection bias in our cohorts because we only recruited patients who underwent RP as initial therapy for localized prostate cancer. Although testing for deviation from HWE in cases has not much meaning for quality control, we still excluded those SNPs from further analyses.

In conclusion, the tagging SNP approach provides comprehensive evidence for the role of the Hippo pathway in prostate cancer progression. The strengths of this study include the discovery-validation study design, the availability of complete clinical information to adjust for potential confounding factors, and the hypothesis-driven approach focused on genetic variants of interest in the Hippo pathway. Although there are differences in clinicopathologic features between the two cohorts included, the utilization of two independent study populations is a particular strength of the study. Given the multiple comparisons being made, the replication of data in an independent population reduced the possibility that significant results might have been caused by chance. The major limitation relates to the sample size in both cohorts, which might limit the power to detect additional significant associations. Although BCR is a relevant clinical end point, prostate cancer mortality should be also explored. In addition, our findings in these homogeneous Taiwanese populations might not be generalizable to other ethnic groups. Overall, unraveling the association between Hippo pathway genes and prostate cancer in this study provides insights into more general mechanisms of disease etiology, and may facilitate the development of novel prognostic biomarkers for prostate cancer. Furthermore, our findings support the development of the Hippo pathway as a potential therapeutic target for prostate cancer.

## Methods

### Patient recruitment and data collection

This study included 458 Taiwanese patients who underwent RP as initial therapy for localized prostate cancer, as described previously^23^,^24^,^25^,^26^. Briefly, the study population consisted of participants from two independent cohorts. The discovery cohort was composed of 246 patients from the National Taiwan University Hospital located in northern Taiwan, and the replication cohort was composed of 212 patients from the Kaohsiung Medical University Hospital, E-Da Hospital, and Kaohsiung Veterans General Hospital, all located in southern Taiwan. Demographic, clinical, and follow-up data were obtained from the medical records. BCR was defined as two consecutive PSA values of at least 0.2 ng/mL^27^,^28^. This study was approved by the Institutional Review Boards of National Taiwan University Hospital, Kaohsiung Medical University Hospital, E-Da Hospital, and Kaohsiung Veterans General Hospital. Written informed consent was obtained from each patient, and the study was carried out in accordance with the approved guidelines.

### SNP selection and genotyping

Genomic DNA was extracted from peripheral blood with the QIAamp DNA Blood Maxi Kit (Qiagen, Valencia, CA, USA) according to the manufacturer's protocol, and stored until the time of study. We utilized a tagging SNP approach to select genetic variants for investigating all the genetic variability in the nine core genes of the Hippo pathway, *MST1*, *STK3*, *LATS1*, *LATS2*, *SAV1*, *MOB1A*, *MOB1B*, *YAP1*, and *WWTR1*. Tagging SNPs were selected using the Tagger algorithm with *r*^2^ ≥ 0.8, and minor-allele frequencies ≥0.2 based on the HapMap population data for Han Chinese in Beijing, China (CHB)^29^,^30^. We identified 75 tagging SNPs, which were genotyped at the National Center for Genome Medicine, Taiwan, using the Sequenom iPLEX matrix-assisted laser desorption/ionization time-of-flight mass-spectrometry technology. For quality control, we randomly selected 10 samples for duplicates, and the concordance rate was >0.99 for all SNPs assayed. Any SNP that failed at Sequenom assay design (n = 6), did not conform to HWE (*P* < 0.001, n = 13), or fell below a genotyping call rate of 0.8 (n = 3), was removed. Thus, a total of 53 SNPs were included for further statistical analyses.

### Statistical analysis

Patient clinicopathologic characteristics were summarized as either the numbers and percentages of patients, or the median and interquartile range of values. Individual SNPs were initially assessed using the Kaplan-Meier analysis with log-rank test for the three genetic models of inheritance: dominant (common homozygotes versus variant allele carrying genotypes), recessive (common allele carrying genotypes versus variant homozygotes), and additive (*P* for trend). Only dominant and additive models were considered if the variant homozygotes were observed in <0.05 of the study population. Multivariate Cox proportional hazards regression analyses were used to assess the effect of each SNP on BCR, with or without adjusting for known prognostic factors, including age, PSA at diagnosis, pathologic Gleason score, and stage, as previously described^24^. The Statistical Package for the Social Sciences software, version 22.0.0 (IBM, Armonk, NY, USA), was used for statistical analyses. A two-sided *P* value of <0.05 was considered statistically significant. Heterogeneity between cohorts was evaluated by Cochran's *χ*^2^-based *Q* statistical test. If the results of the *Q* test were significant, a random-effects model was used to accommodate the diversity; otherwise, the combined HR was estimated using the fixed-effects model.

### Bioinformatics analysis

We used several bioinformatics tools to assess whether rs7827435 or its linked genetic variants were associated with a putative function that might affect patient outcomes. HaploReg v2^31^ and the ENCODE^32^ were used to identify the regulatory potential of the region adjoining the SNPs. The GTEx data were used to identify the correlations between SNPs and prostate tissue-specific gene expression levels^33^. The publicly available cBioPortal for Cancer Genomics^34^ and TCGA datasets for prostate adenocarcinomas^35^ were utilized in order to analyze *STK3* gene expression, DNA methylation, gene copy number, and clinical outcomes.

## Author Contributions

C.Y.H., S.P.H., V.C.L., C.C.Y. and B.Y.B. conceived and designed the experiments. T.Y.C. and S.H.J. performed the experiments and analyzed the data. All authors wrote, reviewed, and approved submission of the paper.

## Supplementary Material

Supplementary Information Supplementary Table 1

## Figures and Tables

**Table 1:** Clinical characteristics of study cohorts

Characteristic	Discovery	Replication	Combined	*P*^a^
Patients, n	246	212	458	
Age at diagnosis				0.303
Median, y (IQR)	65 (61–69)	68 (62–71)	66 (61–70)	
≤65	134 (54.5)	77 (36.3)	211 (46.1)	
>65	112 (45.5)	135 (63.7)	247 (53.9)	
PSA at diagnosis				<0.001
Median, ng/mL (IQR)	10.1 (6.7–15.8)	12.6 (7.6–19.8)	11.1 (7.1–17.5)	
≤10	116 (49.4)	81 (39.7)	197 (44.9)	
>10	119 (50.6)	123 (60.3)	242 (55.1)	
Pathologic Gleason score, n (%)				<0.001
≤7	217 (89.7)	175 (82.9)	392 (86.5)	
>7	25 (10.3)	36 (17.1)	61 (13.5)	
Pathologic stage, n (%)				<0.001
T1/T2	173 (72.4)	130 (61.3)	303 (67.2)	
T3/T4/N1	66 (27.6)	82 (38.7)	148 (32.8)	
Recurrence	75 (30.5)	109 (51.4)	184 (40.2)	
Median follow-up time^b^, mo (95% CI)	50 (45–55)	60 (56–64)	54 (50–58)	

Abbreviations: IQR, interquartile range; PSA, prostate-specific antigen; CI, confidence interval.

^a^*P* value was calculated by the log-rank test for recurrence in combined 458 patients.

^b^Median follow-up time and 95% CIs were estimated with the reverse Kaplan-Meier method.

**Table 2:** Association of *STK3* rs7827435 with BCR in localized prostate cancer patients treated with RP

	Univariate analysis										Multivariate analysis^a^					
Gene SNP	Discovery				Replication				Combined		Discovery		Replication		Combined	
Genotype	No BCR	BCR	HR (95% CI)	*P*	No BCR	BCR	HR (95% CI)	*P*	HR (95% CI)	*P*	HR (95% CI)	*P*	HR (95% CI)	*P*	HR (95% CI)	*P*
*STK3* rs7827435																
AA	40	23	1.00		25	29	1.00		1.00		1.00		1.00		1.00	
AT	87	38	0.80 (0.47–1.34)	0.387	53	59	1.10 (0.70–1.72)	0.690	0.96 (0.68–1.36)	0.83	0.93 (0.54–1.59)	0.785	0.90 (0.57–1.41)	0.635	0.91 (0.64–1.29)	0.61
TT	40	12	**0.49 (0.25–0.99)**	**0.048**	24	16	0.60 (0.32–1.10)	0.097	**0.55 (0.34–0.86)**	**0.01**	**0.43 (0.20–0.89)**	**0.024**	**0.49 (0.26–0.93)**	**0.028**	**0.46 (0.29–0.76)**	**0.002**
AT/TT vs AA			0.69 (0.42–1.14)	0.147			0.93 (0.60–1.42)	0.725	0.82 (0.59–1.13)	0.23	0.73 (0.44–1.21)	0.220	0.76 (0.49–1.18)	0.220	0.75 (0.54–1.04)	0.08
TT vs AA/AT			0.57 (0.31–1.07)	0.079			**0.56 (0.33–0.96)**	**0.034**	**0.56 (0.38–0.84)**	**0.005**	**0.45 (0.23–0.87)**	**0.018**	**0.53 (0.30–0.93)**	**0.026**	**0.49 (0.32–0.76)**	**0.001**
Trend			**0.72 (0.52–1.00)**	**0.048**			0.81 (0.62–1.07)	0.133	**0.77 (0.63–0.95)**	**0.01**	**0.70 (0.50–0.97)**	**0.030**	**0.73 (0.55–0.98)**	**0.033**	**0.72 (0.58–0.89)**	**0.003**

Abbreviations: BCR, biochemical recurrence; RP, radical prostatectomy; SNP, single nucleotide polymorphism; HR, hazard ratio; CI, confidence interval; PSA, prostate-specific antigen.

^a^Adjusted by age, PSA at diagnosis, pathologic Gleason score, and pathologic stage.

*P* < 0.05 are in boldface.
