# Supplementary material for: Genetic variants in the Hippo pathway predict biochemical recurrence after radical prostatectomy for localized prostate cancer
Source: Sci Rep. 2015 Feb 24;5:8556. doi: 10.1038/srep08556 (PMC4338420; doi:10.1038/srep08556)
Supplement: Supplementary Information — Supplementary Table 1 [file srep08556-s1.doc]

**Genetic variants in the Hippo pathway predict biochemical recurrence after radical prostatectomy for localized prostate cancer**

Chao-Yuan Huang^1,†^, Shu-Pin Huang^2,3,†^, Victor C. Lin^4,5^, Chia-Cheng Yu^6,7,8^, Ta-Yuan Chang^9^, Shin-Hun Juang^10^ & Bo-Ying Bao^10,11,12,*^

^1^Department of Urology, National Taiwan University Hospital, College of Medicine, National Taiwan University, Taipei, Taiwan, ^2^Department of Urology, Kaohsiung Medical University Hospital, Kaohsiung, Taiwan, ^3^Department of Urology, Faculty of Medicine, College of Medicine, Kaohsiung Medical University, Kaohsiung, Taiwan, ^4^Department of Urology, E-Da Hospital, Kaohsiung, Taiwan, ^5^School of Medicine for International Students, I-Shou University, Kaohsiung, Taiwan, ^6^Division of Urology, Department of Surgery, Kaohsiung Veterans General Hospital, Kaohsiung, Taiwan, ^7^Department of Urology, School of Medicine, National Yang-Ming University, Taipei, Taiwan, ^8^Department of Pharmacy, Tajen University, Pingtung, Taiwan, ^9^Department of Occupational Safety and Health, China Medical University, Taichung, Taiwan, ^10^Department of Pharmacy, China Medical University, Taichung, Taiwan, ^11^Sex Hormone Research Center, China Medical University Hospital, Taichung, Taiwan, ^12^Department of Nursing, Asia University, Taichung, Taiwan

^†^These authors contributed equally to this work.

^*^Correspondence and requests for materials should be addressed to B.Y.B. (bao@mail.cmu.edu.tw)

Supplementary Table 1 | Genotyped SNPs and the *P* values of their association with BCR after RP

| Gene | SNP ID | Chromosome | Position | Minor allele | MAF | | | | BCR | | |
| --- | --- | --- | --- | --- | --- | --- | --- | --- | --- | --- | --- |
|  |  |  |  |  | This study TWN | HapMap CHB | HapMap CEU | HapMap YRI | Additive | Dominant | Recessive |
| *MOB1A* | rs1620482 | chr2 | 74240734 | C | 0.201 | 0.304 | 0.385 | 0.211 | 0.594 | 0.973 | 0.119 |
| *MOB1A* | rs828891 | chr2 | 74243456 | G | 0.385 | 0.315 | 0.487 | 0.093 | 0.994 | 0.754 | 0.701 |
| *MOB1A* | rs7596645 | chr2 | 74258627 | T | 0.398 | 0.387 | 0.106 | 0.204 | 0.767 | 0.916 | 0.668 |
| *WWTR1* | rs6806548 | chr3 | 150722141 | T | 0.335 | 0.315 | 0.179 | 0.438 | 0.068 | 0.097 | 0.209 |
| *WWTR1* | rs9289790 | chr3 | 150725647 | A | 0.248 | 0.262 | 0.580 | 0.500 | 0.645 | 0.608 | 0.918 |
| *WWTR1* | rs10935766 | chr3 | 150727476 | C | 0.190 | 0.211 | 0.292 | 0.292 | 0.611 | 0.327 | - |
| *WWTR1* | rs16861941 | chr3 | 150729891 | A | 0.228 | 0.209 | 0.051 | - | 0.515 | 0.540 | 0.702 |
| *WWTR1* | rs7616772 | chr3 | 150730329 | A | 0.267 | 0.262 | 0.353 | 0.314 | 0.285 | 0.123 | 0.690 |
| *WWTR1* | rs4681183 | chr3 | 150738168 | T | 0.298 | 0.322 | 0.250 | 0.708 | 0.389 | 0.280 | 0.964 |
| *WWTR1* | rs2278477 | chr3 | 150743505 | C | 0.470 | 0.578 | 0.500 | 0.717 | 0.236 | 0.151 | 0.661 |
| *WWTR1* | rs9836653 | chr3 | 150749361 | C | 0.202 | 0.202 | 0.593 | 0.159 | 0.877 | 0.603 | - |
| *WWTR1* | rs2043550 | chr3 | 150753475 | C | 0.457 | 0.341 | - | 0.059 | 0.974 | 0.624 | 0.601 |
| *WWTR1* | rs16861985 | chr3 | 150754194 | G | 0.161 | 0.233 | 0.000 | 0.133 | 0.082 | 0.101 | - |
| *WWTR1* | rs16861987 | chr3 | 150755047 | T | 0.308 | 0.345 | 0.146 | 0.195 | 0.058 | 0.457 | **0.001** |
| *WWTR1* | rs4681526 | chr3 | 150767122 | C | 0.198 | 0.220 | 0.500 | 0.212 | 0.169 | 0.156 | - |
| *WWTR1* | rs12632407 | chr3 | 150769924 | A | 0.275 | 0.367 | 0.858 | 0.675 | 0.211 | 0.218 | - |
| *WWTR1* | rs7652697 | chr3 | 150775199 | T | 0.310 | 0.298 | 0.265 | 0.395 | 0.510 | 0.677 | 0.445 |
| *WWTR1* | rs16862023 | chr3 | 150807394 | T | 0.226 | 0.238 | 0.000 | 0.183 | 0.800 | 0.449 | 0.391 |
| *WWTR1* | rs16862054 | chr3 | 150822102 | G | 0.225 | 0.238 | 0.000 | 0.000 | 0.419 | 0.739 | 0.178 |
| *WWTR1* | rs1561026 | chr3 | 150834322 | C | 0.296 | 0.256 | 0.733 | 0.788 | 0.315 | 0.188 | 0.923 |
| *WWTR1* | rs9820339 | chr3 | 150841229 | C | 0.301 | 0.375 | 0.588 | 0.155 | 0.794 | 0.718 | 0.995 |
| *WWTR1* | rs9820948 | chr3 | 150844585 | C | 0.454 | 0.544 | 0.742 | 0.800 | 0.668 | 0.890 | 0.558 |
| *WWTR1* | rs9821781 | chr3 | 150845095 | G | 0.485 | 0.458 | 0.314 | 0.345 | 0.299 | 0.157 | 0.793 |
| *WWTR1* | rs2117754 | chr3 | 150846198 | G | 0.342 | 0.333 | 0.376 | 0.248 | 0.164 | 0.104 | 0.718 |
| *WWTR1* | rs6779455 | chr3 | 150847163 | C | 0.391 | 0.387 | 0.513 | 0.367 | 0.309 | 0.770 | 0.123 |
| *WWTR1* | rs13325622 | chr3 | 150856070 | T | 0.436 | 0.387 | 0.588 | 0.496 | 0.328 | 0.134 | 0.983 |
| *WWTR1* | rs6783790 | chr3 | 150857979 | A | 0.492 | 0.542 | 0.403 | 0.320 | 0.166 | 0.820 | **0.038** |
| *LATS1* | rs9393175 | chr6 | 150040416 | A | 0.162 | 0.235 | 0.662 | 0.879 | 0.782 | 0.981 | - |
| *LATS1* | rs17088243 | chr6 | 150041480 | G | 0.166 | 0.202 | 0.000 | 0.000 | **0.007** | **0.014** | - |
| *STK3* | rs16892236 | chr8 | 99544748 | G | 0.449 | 0.512 | 0.367 | 0.009 | 0.894 | 0.567 | 0.400 |
| *STK3* | rs7008395 | chr8 | 99560087 | A | 0.130 | 0.208 | 0.062 | 0.018 | 0.813 | 0.987 | - |
| *STK3* | rs4388428 | chr8 | 99570083 | T | 0.496 | 0.536 | 0.415 | 0.389 | 0.394 | 0.300 | 0.699 |
| *STK3* | rs7815227 | chr8 | 99573246 | A | 0.460 | 0.367 | 0.608 | 0.576 | 0.649 | 0.799 | 0.606 |
| *STK3* | rs7818010 | chr8 | 99646219 | T | 0.468 | 0.522 | 0.242 | 0.200 | 0.512 | 0.156 | 0.658 |
| *STK3* | rs12550012 | chr8 | 99709224 | C | 0.397 | 0.446 | 0.111 | 0.000 | 0.357 | 0.172 | 0.939 |
| *STK3* | rs11785245 | chr8 | 99743475 | T | 0.495 | 0.344 | 0.664 | 0.650 | 0.099 | 0.405 | 0.066 |
| *STK3* | rs10112149 | chr8 | 99819660 | C | 0.368 | 0.295 | 0.387 | 0.088 | 0.324 | 0.540 | 0.287 |
| *STK3* | rs7827435 | chr8 | 99852202 | T | 0.473 | 0.356 | 0.642 | 0.733 | **0.046** | 0.142 | 0.073 |
| *STK3* | rs2515219 | chr8 | 99893226 | G | 0.274 | 0.344 | 0.292 | 0.225 | **0.020** | **0.006** | 0.700 |
| *YAP1* | rs10895257 | chr11 | 101491854 | A | 0.310 | 0.364 | 0.217 | - | 0.206 | 0.057 | 0.649 |
| *YAP1* | rs1426398 | chr11 | 101493271 | C | 0.304 | 0.307 | 0.533 | 0.375 | 0.821 | 0.981 | 0.654 |
| *YAP1* | rs11225138 | chr11 | 101499108 | C | 0.221 | 0.196 | 0.102 | 0.009 | 0.354 | 0.211 | - |
| *YAP1* | rs10895264 | chr11 | 101515891 | T | 0.391 | 0.405 | 0.350 | 0.592 | 0.237 | 0.148 | 0.801 |
| *YAP1* | rs9787758 | chr11 | 101530181 | C | 0.257 | 0.238 | 0.496 | 0.035 | 0.344 | 0.404 | 0.496 |
| *YAP1* | rs7925543 | chr11 | 101587087 | C | 0.272 | 0.200 | 0.045 | 0.094 | 0.202 | 0.513 | 0.055 |
| *YAP1* | rs10895276 | chr11 | 101588905 | T | 0.405 | 0.420 | 0.325 | 0.408 | 0.498 | 0.603 | 0.552 |
| *YAP1* | rs11225170 | chr11 | 101598674 | C | 0.343 | 0.387 | 0.353 | 0.201 | 0.320 | 0.254 | 0.740 |
| *LATS2* | rs688663 | chr13 | 20452217 | T | 0.459 | 0.410 | 0.863 | 0.673 | 0.880 | 0.571 | 0.674 |
| *LATS2* | rs9552332 | chr13 | 20503560 | G | 0.433 | 0.542 | 0.034 | - | 0.785 | 0.597 | 0.889 |
| *LATS2* | rs4770094 | chr13 | 20510791 | A | 0.382 | 0.375 | 0.726 | 0.319 | 0.755 | 0.640 | 0.983 |
| *LATS2* | rs9509508 | chr13 | 20523473 | T | 0.440 | 0.446 | 0.726 | 0.128 | 0.945 | 0.829 | 0.897 |
| *SAV1* | rs12898086 | chr14 | 50184467 | T | 0.456 | 0.512 | 0.881 | 0.929 | 0.150 | 0.468 | 0.099 |
| *SAV1* | rs923908 | chr14 | 50193345 | G | 0.245 | 0.315 | 0.540 | 0.757 | 0.825 | 0.839 | 0.879 |

Abbreviations: SNP, single nucleotide polymorphism; BCR, biochemical recurrence; RP, radical prostatectomy; MAF, minor allele frequency; TWN, Taiwanese; CHB, Han Chinese in Beijing, China; CEU, Utah residents with ancestry from northern and western Europe; YRI, Yoruba in Ibadan, Nigeria.

*P* values for log-rank test.

*P* < 0.05 is in boldface.
